# Phase-specific multimodal biomarkers enable explainable assessment of upper limb dysfunction in chronic stroke

**DOI:** 10.3389/fnins.2025.1737407

**Published:** 2025-12-01

**Authors:** Lei Li, Junhong Wang, Jingcheng Chen, Shaoming Sun, Wei Peng

**Affiliations:** 1 Hefei Institutes of Physical Science, Chinese Academy of Sciences, Hefei, China; 2 University of Science and Technology of China, Hefei, China; 3 Institute of Artificial Intelligence, Hefei Comprehensive National Science Center, Hefei, China; 4 CAS Hefei Institute of Technology Innovation, Hefei, China

**Keywords:** stroke, upper limb motor impairments, musculoskeletal modeling, explainable artificial intelligence, phase-specific multimodal data

## Abstract

**Background:**

Objective and precise assessment of upper limb dysfunction post-stroke is critical for guiding rehabilitation. While promising, current methods using wearable sensors and machine learning (ML) often lack interpretability and neglect underlying, phase-specific kinetic deficits (e.g., muscle forces and joint torques) within functional tasks. This study aimed to develop and validate an explainable assessment framework that leverages musculoskeletal kinetic modeling to extract phase-specific, multimodal (kinematic and kinetic) biomarkers to assess upper limb dysfunction in chronic stroke.

**Methods:**

Sixty-five adults with chronic stroke and 20 healthy controls performed a standardized hand-to-mouth (HTM) task. Stroke participants were allocated to a model-development cohort (n = 47) and an independent test cohort (*n* = 18). Using IMU and sEMG data, we employed musculoskeletal modeling to extract phase-specific kinematic (e.g., inter-joint coordination, trunk displacement) and kinetic (e.g., mechanical work, smoothness, co-contraction index) biomarkers from four task phases. A Lasso regression model was trained to predict FMA-UL scores, validated via 5-fold cross-validation and the independent test cohort. Explainable AI (SHAP) was used to identify key predictive features.

**Results:**

Compared with controls, patients showed phase-specific alterations including greater trunk displacement and reduced inter-joint coordination and mechanical work (all *p* < 0.05). The Lasso model achieved strong performance in internal validation (*R*^2^ = 0.932; MAE = 0.799) and generalized well to the independent test cohort (*R*^2^ = 0.881; MAE = 0.954). SHAP identified trunk displacement in phase 2 (TD_2), elbow–shoulder coordination in phase 3 (IC_elb_elv_3), and trunk displacement in phase 3 (TD_3) as dominant predictors; larger trunk displacement contributed negatively to predicted FMA-UL scores.

**Conclusion:**

Integrating phase-specific multimodal biomarkers with explainable ML yields an interpretable upper-limb dysfunction. By highlighting phase-specific kinetic and kinematic targets (e.g., trunk compensation and inter-joint coordination), the framework supports individualized, precision rehabilitation.

## 1 Introduction

Stroke is the leading cause of long-term disability among adults worldwide, with more than 60% of patients experiencing upper limb motor impairments during both the acute and chronic phases of recovery (Geyh et al., 2004). These impairments severely restrict patients’ ability to perform activities of daily living (ADL) and significantly reduce their quality of life (Krakauer, 2005). Currently, rehabilitation training is the most widely recognized and effective intervention for post-stroke functional impairment (Pomeroy et al., 2011; Sathian et al., 2011). It promotes functional reorganization and improves daily activity performance through repetitive motor training of the affected upper limb (Dobkin, 2004). However, this process may take months or even years (Naghdi et al., 2010). During this period, clinicians must regularly assess the severity of motor impairment or functional status to monitor rehabilitation progress, evaluate treatment effectiveness, and develop individualized rehabilitation programs (Cooke et al., 2010). The Fugl-Meyer Assessment for the Upper Limb (FMA-UL) is considered the gold-standard clinical tool for such evaluations (Frey et al., 2011; Bernhardt et al., 2017). Nevertheless, the test is time-consuming, highly subjective, and requires patients to perform a series of predefined movements, which do not reflect upper-limb use in daily life (Stewart and Cramer, 2013).

Previous research has demonstrated a close relationship between daily-life activity performance and traditional clinical functional assessments, such as the FMA-UL scale (McCrea et al., 2002; Lang et al., 2013; Ingram et al., 2021). This finding suggests that daily activity monitoring may provide valuable, ecologically valid information that complements conventional evaluation methods. Recently, wearable sensors such as inertial measurement units (IMUs) and surface electromyography (sEMG), in combination with machine learning approaches, have provided promising opportunities to assess upper-limb motor function during real-world daily activities (Wang et al., 2024). For example, Murphy MA et al. analyzed a drinking task involving reaching and grasping movements using three-dimensional kinematic features to distinguish stroke severity levels based on FMA-UL scores (Murphy et al., 2010). Oubre et al. used one-to-two-minute random voluntary upper-limb movements and applied unsupervised clustering and supervised regression models to estimate FMA-UL scores from features extracted from these sub movements (Oubre et al., 2020). Adans-Dester et al. proposed machine learning-based algorithms to derive clinical score estimates from wearable sensor data collected during functional motor tasks, demonstrating strong agreement with clinician-rated FMA-UL scores (Adans-Dester et al., 2020). Razfar et al. proposed a PSA-NMF clustering algorithm that used camera-based and wearable sensor-based features of reaching movements to cluster stroke survivors according to FMA-UL severity levels (Razfar et al., 2023). Ye et al. who developed a backpropagation neural network (BPNN) model based on sEMG to automatically map FMA-UL and MAS scores (Ye et al., 2021). This approach, leveraging deep learning networks to automatically extract features from complex bioelectrical signals for classification, has demonstrated potential in various biomedical fields (Liu et al., 2020, 2025a). However, while these black-box models achieve high predictive accuracy, they lack clinical interpretability, limiting their application in evidence-based rehabilitation decision-making (Sorayaie Azar et al., 2024).

The essence of post-stroke motor impairment is multidimensional neuromuscular dysfunction, involving weakness, spasticity, abnormal synergies, and disordered motor control (Krakauer, 2005; Langhorne et al., 2009; Raghavan, 2015). These mechanisms are not only reflected in joint kinematics but are also fundamentally rooted in kinetics, such as joint torques and muscle forces (Buma et al., 2013; Raghavan, 2015). Compared with purely kinematic measures, kinetic analysis provides more direct insights into the underlying neuromuscular pathophysiology (Kamper et al., 2003; Langhorne et al., 2009). Musculoskeletal dynamic modeling and inverse dynamics analysis, particularly when combined with wearable data from IMU and sEMG (Akhundov et al., 2022; Giarmatzis et al., 2022), enable quantitative estimation of internal biomechanical loads (e.g., joint torques, muscle forces) during functional tasks. For example, Ang et al. used musculoskeletal modeling to analyze spasticity during passive elbow motion (Ang et al., 2018), and Tahmid S et al. highlighted EMG-driven modeling as a valuable tool for explaining muscle dysfunction after stroke (Tahmid et al., 2022).

Although the aforementioned studies have laid an important foundation for objective, kinetics-based assessment of upper limb motor impairments, further exploration of the internal subphase structure within complex ADL tasks is still needed. In reality, ADL tasks such as the hand-to-mouth task - widely adopted in kinematic and kinetic studies for its universality, standardized structure, and strong representativeness of daily-life movements (Saes et al., 2022; Ota et al., 2023; Huang et al., 2024; Unger et al., 2024) - typically consist of multiple functionally distinct fundamental movement primitives arranged in a specific sequence (Giszter, 2015), such as “Reach” and “Transport” (Schwarz et al., 2022). While similar to the drinking task, the HTM task does not include grasping as an additional variable (Huang et al., 2024). This allows us to more purely analyze the core motor chain from reaching to transport (i.e., shoulder/elbow/trunk coordination). Several studies have attempted to characterize such phase-specific movement patterns. Murphy et al. divided the drinking task into five subphases and extracted kinematic features that effectively differentiated patients with moderate and mild post-stroke upper-limb impairments (Murphy et al., 2010). Repnik et al. examined variations in five kinematic variables across different subphases of upper-limb movements in individuals’ post-stroke (Repnik et al., 2018), thereby illustrating the feasibility of using motion-based metrics to distinguish between participant groups. These findings suggest that movement primitives can serve as finer-grained and more stable motor control modules. The strategy of decomposing complex tasks into fine-grained analytical units has been shown to improve both accuracy and interpretability in other neuroengineering fields (Liu et al., 2025b). However, current evidence remains predominantly kinematic in nature, with limited investigation into muscle force generation or kinetic characteristics across distinct subphases. Ignoring this phase-specific nature of tasks may not only obscure phase-specific, critical pathophysiological information but also limit the clinical interpretability of assessment results for guiding personalized, phase-targeted rehabilitation interventions (Jackson et al., 2023).

Explainable machine learning has recently gained traction in medical and rehabilitation research, emphasizing not only prediction accuracy but also model transparency and interpretability (Apostolidis et al., 2023; Gurmessa and Jimma, 2023). This pursuit of interpretability is cross-disciplinary. In computer vision, for example, XAI methods are also used to balance model faithfulness against plausibility to build user trust (Liu et al., 2024). For clinical adoption, the explainability of predictions is paramount. Methods like SHAP and feature importance ranking enable researchers to uncover the direction and magnitude of contributions from different kinetic parameters to predictions, thereby aiding in understanding the underlying physiological mechanisms (Martínez-Cid et al., 2025). Combining phase-specific kinetic modeling with explainable machine learning can enhance the identification accuracy of upper limb dysfunction in stroke patients while ensuring results are clinically interpretable and translatable.

This study aims to develop and rigorously validate a novel framework that assesses upper limb dysfunction by extracting phase-specific, multimodal biomarkers (kinematics and kinetics) from the standardized hand-to-mouth (HTM) task using wearable sensors (IMU/sEMG) and musculoskeletal modeling. The primary objectives were: (1) To identify phase-specific biomechanical alterations by comparing kinetic and kinematic features between chronic stroke patients and healthy controls; (2) To develop an ML model capable of accurately predicting upper limb dysfunction (quantified by FMA-UL scores), and critically, to validate its generalizability using an independent test cohort; and (3) To employ explainable AI (SHAP) to identify which key biomarkers drive the predictions, thereby explaining how these biomechanical alterations contribute to the overall dysfunction score. We hypothesized that this phase-specific kinetic approach would not only yield high predictive accuracy but also provide clinically interpretable insights, identifying new quantitative targets for personalized rehabilitation.

## 2 Materials and methods

### 2.1 Participants

We enrolled 65 adults with chronic stroke and 20 age-matched healthy controls. Stroke participants were allocated to a model-development cohort (*n* = 47) and an independent test cohort (*n* = 18), with the test cohort held out throughout model development and tuning. All participants were recruited under identical eligibility criteria. Inclusion criteria were: first-ever unilateral stroke confirmed by neuroimaging with persistent motor impairment of the paretic upper limb; chronic stage (≥ 6 months post-stroke); sufficient voluntary movement to complete the hand-to-mouth task, operationalized as active elbow flexion ≥ 30°; ability to sit unsupported and follow verbal commands; and adequate cognition (MMSE ≥ 24). Exclusion criteria were: recurrent or bilateral stroke; marked upper-limb spasticity (Modified Ashworth Scale > 3); musculoskeletal deformity or pain limiting upper-limb motion; severe neglect, aphasia, or apraxia compromising task performance; and cardiovascular or orthopedic conditions judged unsafe or likely to interfere with standardized testing.

### 2.2 Experimental protocol and data collection

A standardized hand-to-mouth (HTM) task (Cai et al., 2021), known for good test–retest reliability, was used as the primary functional assessment to measure upper limb motor ability during drinking, eating, and face-approach activities. The task required participants to reach from a starting position on a table to touch a marker, then lift their hand to touch their lips with the thumb, return to touch the starting marker, and finally return to the initial position. The marker was positioned 30 cm from the table edge at the body midline, calibrated to approximately 80% of individual arm length. Participants sat on an adjustable-height chair with their back against the chair back, but sitting posture was not rigidly constrained to allow necessary compensatory movements. The initial posture required the upper arm hanging naturally, elbow flexed approximately 90°, and the tested hand resting palm-down on the table. All movements were performed at a self-selected pace to better reflect natural ADL performance and avoid inducing abnormal compensatory patterns that may arise from speed-focused instructions. Only the paretic upper limb was tested in the stroke group; the dominant hand was tested in the control group. Each participant completed five task trials; the average of the middle three trials was used for final analysis. Adequate rest was provided between trials to avoid muscle fatigue.

**Figure 1:**
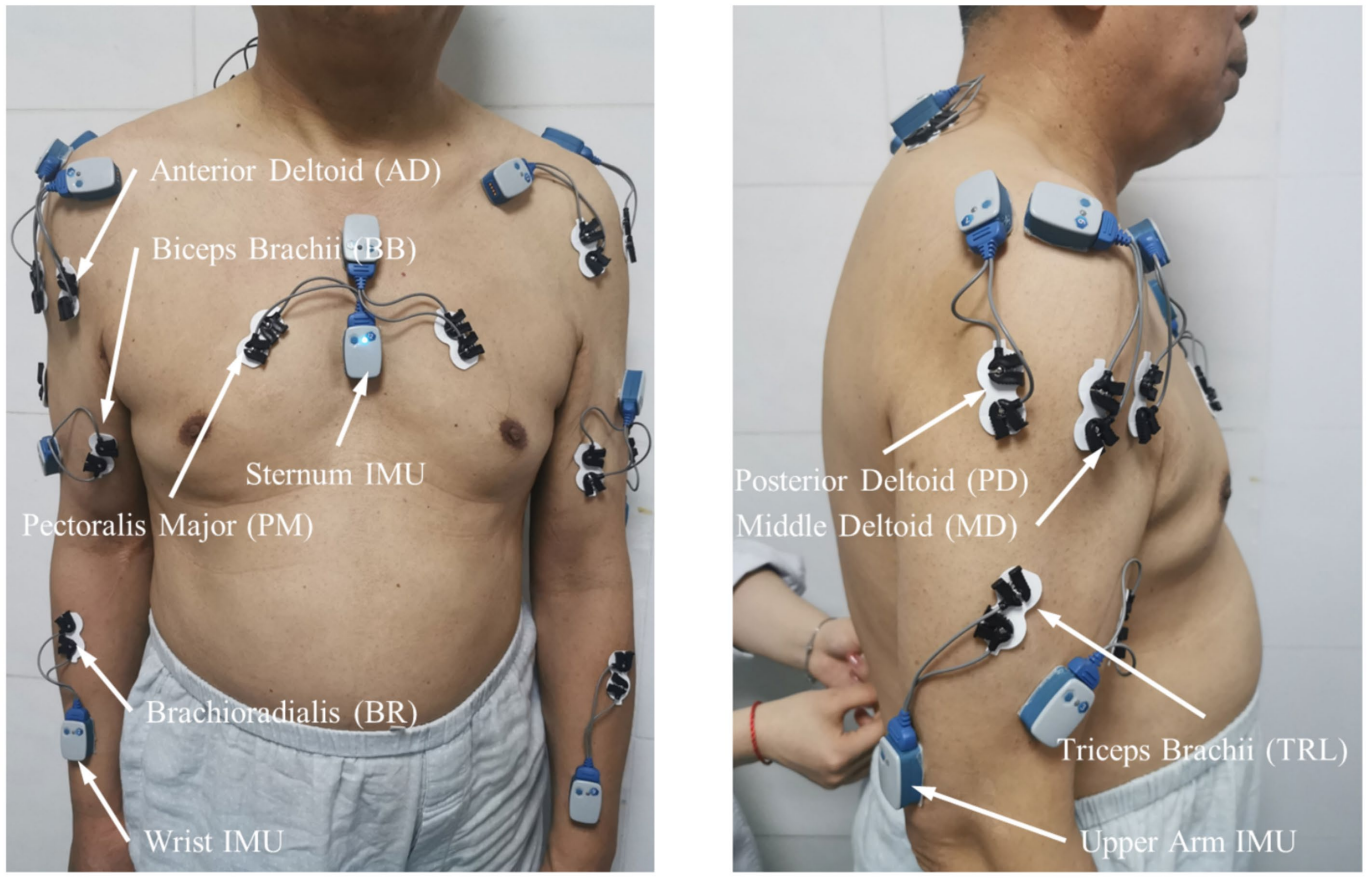
Schematic placement of sensors used for data collection. Three inertial measurement units (IMUs) were attached to the sternum, upper arm, and wrist to capture trunk and upper-limb kinematics. Surface EMG electrodes were placed over seven muscles of the paretic upper limb that were included in the present analysis: Brachioradialis (BR), Biceps Brachii (BB), Triceps Brachii Lateral Head (TRL), Anterior Deltoid (AD), Middle Deltoid (MD), Posterior Deltoid (PD), and Pectoralis Major (PM). One additional electrode over the upper trapezius, whose data were not used in the present analyses, is also visible.

All movements were completed during a single laboratory visit, with synchronous acquisition of surface electromyography (sEMG) and inertial measurement unit (IMU) data. sEMG signals were recorded at 2000 Hz using a wearable system (Noraxon USA, Inc., Scottsdale, AZ, United States) from seven upper limb muscles: Brachioradialis (BR), Biceps Brachii (BB), Triceps Brachii Lateral Head (TRL), Anterior Deltoid (AD), Middle Deltoid (MD), Posterior Deltoid (PD), and Pectoralis Major (PM). Bipolar Ag-AgCl circular surface electrodes, spaced 2 cm apart, were placed longitudinally over the muscle belly center according to recommendations from the International Society of Electrophysiology and Kinesiology (ISEK), following shaving and alcohol cleansing. IMU modules recorded data at 400 Hz and were fixed to the sternum, upper arm, and wrist. Data were wirelessly transmitted via Bluetooth to a host computer for synchronized storage and processing. The placement of sensors and electrodes is illustrated in Figure 1.

### 2.3 Data analysis procedure

The analysis pipeline commenced with preprocessing of the raw sensor data. Joint kinematics were derived from the raw IMU data (angular velocity and acceleration) using established sensor fusion and calibration procedures (Nazarahari and Rouhani, 2021). Concurrently, raw sEMG signals were band-pass filtered, full-wave rectified, and low-pass filtered to create linear envelopes; these envelopes were then normalized to the peak value recorded during the task cycles (Lloyd and Besier, 2003). These processed kinematic and sEMG data served as the primary inputs for the subsequent musculoskeletal modeling pipeline.

Prior to kinetic analysis, the open-source OpenSim (v4.3) upper limb musculoskeletal model (Seth et al., 2019) was scaled to match each participant’s anthropometry. This critical step utilized participant-specific measurements (e.g., height, weight, and segment lengths derived from IMU positions) to adjust the model’s body segment parameters and muscle-tendon paths, thereby mitigating potential anthropometric bias. Once scaled, kinematic data from IMUs and preprocessed sEMG signals were input into the model. The analysis was performed using the Calibrated EMG-Informed Neuromuscular Modeling toolbox (CEINMS) (CEINMS: a toolbox to investigate the influence of different neural control solutions on the prediction of muscle excitation and joint moments during dynamic motor tasks Pizzolato et al., 2015). The pipeline commenced with the OpenSim Scaling tool, followed by Inverse Kinematics (IK) to obtain joint angles, and then Inverse Dynamics (ID) to calculate net joint torques. Crucially, to enhance the subject-specific physiological accuracy, the CEINMS calibration procedure was performed. This standard step iteratively optimized key musculoskeletal parameters (e.g., tendon slack length) to minimize the error between the EMG-driven model-predicted joint torques and the ID-calculated net joint torques. Subsequently, this fully calibrated, subject-specific model was implemented in an EMG-informed mode: recorded EMG signals directly informed the activations of the corresponding measured muscles, while activations for unmeasured muscles were estimated through optimization (using the standard static optimization algorithm in the CEINMS toolbox, which aims to minimize the sum of squared muscle activations). This EMG-driven approach, which incorporates muscle activation-force generation dynamics models, was employed to estimate force output time-series for major muscles during each task. The entire kinetic computation pipeline followed the standard, validated CEINMS workflow (CEINMS: a toolbox to investigate the influence of different neural control solutions on the prediction of muscle excitation and joint moments during dynamic motor tasks Pizzolato et al., 2015; Seth et al., 2019; Tahmid et al., 2022), ensuring scientific rigor and reproducibility.

The HTM task was segmented into four consecutive subphases: Reach to object phase (Phase 1), Transfer to mouth phase (Phase 2), Transfer to object phase (Phase 3) and Return to start phase (Phase 4). Phase segmentation was based on 3D spatial trajectory and velocity characteristics recorded by the wrist IMU. Phase segmentation was performed by identifying key kinematic landmarks within the 3D velocity profile recorded by the wrist IMU (e.g., movement onset, velocity peaks, and movement offset), following established protocols for HTM task analysis (Schwarz et al., 2022; Huang et al., 2024). This algorithmic segmentation was then visually verified by trained researchers to ensure precise temporal delineation of the movement sequence, providing a clear structure for subsequent phase-specific feature extraction.

Based on the IMU- and sEMG-driven musculoskeletal model, key kinetic features were systematically calculated and extracted during each phase of the movement, providing structured input data for subsequent ML-based multi-phase sequence regression analysis and FMA-UL scores prediction. Joint kinematic features were extracted based on the upper limb degrees of freedom defined in the OpenSim model, where elb denotes elbow flexion/extension, elv denotes shoulder elevation/depression, rot denotes shoulder internal/external rotation, and fle denotes shoulder flexion/extension. For each HTM task phase, five categories of parameters were calculated:

1. Mechanical work (W) (Seth et al., 2019; Wright et al., 2020) performed by joints and muscles was calculated by integrating muscle power time-series, quantifying an individual’s force output contribution during task execution.
2. The smoothness of joint moments and individual muscle forces over time was assessed using the Spectral Arc Length (SPARC) method (Balasubramanian et al., 2015; Mohamed Refai et al., 2021), reflecting stability and smoothness of force output control.
3. Co-contraction indices (CCI) (Bandini et al., 2023) were calculated for four agonist/antagonist or synergistic muscle pairs (AD/PD, TRL/BB, MD/PM, TRL/BR) by analyzing the time-series overlap integral of their force profiles. This quantifies muscle co-activation levels during the motor task, revealing synergistic and stiffness regulation capabilities.
4. Interjoint coordination (IC) (Murphy et al., 2010) was assessed by calculating the correlation coefficient between shoulder and elbow joint angles. A value closer to 1.0 indicates stronger correlation and tighter coupling of motion between the two joints.
5. To identify compensatory trunk movements arising from upper limb impairment, trunk displacement magnitude (TD) (Repnik et al., 2018) was quantified using the norm of the 3D displacement (x, y, z) recorded by the sternum IMU, measuring the overall trunk movement amplitude during the task.

The average value of the middle three trials for each participant was used for statistical analysis. Feature data were first tested for normality. For normally distributed features, independent samples t-tests were used to compare differences between the healthy and stroke groups. For non-normally distributed features, the Mann–Whitney U test was used. Descriptive statistics: normally distributed features are reported as mean ± standard deviation; non-normally distributed features are reported as median (interquartile range, IQR). All feature labels and abbreviations (e.g., TD_2, S_elb_1) used in this study are defined in the note of Table 1. To account for multiple comparisons, all *p* values were adjusted using the Benjamini-Hochberg False Discovery Rate (FDR) procedure. Statistical significance was set at an FDR-adjusted *p* < 0.05. To quantify the magnitude of group differences, effect sizes were calculated using Cohen’s d. Statistical analyses were performed using SPSS 24.0 (IBM, United States).

**Table 1:** Group comparison of features between healthy and patient participants.

Feature	Healthy	Patient	*p* value	Cohen’s d
TD_2	5.44 (4.58)	8.90 (14.93)	0.036	0.680
TD_3	7.00 (8.68)	11.91 (21.43)	0.012	0.682
TD_4	3.11 (3.34)	10.62 (15.13)	0.015	0.612
IC_elb_elv_2	0.96 (0.04)	0.91 (0.19)	0.015	−0.502
IC_elb_elv_3	0.94 (0.07)	0.85 (0.15)	0.008	−0.625
IC_elb_elv_4	−0.11 ± 0.53	0.24 ± 0.46	0.033	0.708
W_elb_2	1.23 (0.63)	0.81 (0.43)	0.017	−0.931
W_elb_3	−1.14 (0.44)	−0.80 (0.32)	0.017	0.866
W_BB_2	1.17 (0.97)	0.72 (0.64)	0.017	−0.805
W_BB_3	−1.69 ± 0.85	−1.20 ± 0.54	0.023	0.755
W_TRI_3	4.96 ± 1.78	3.99 ± 1.22	0.036	−0.689
S_elb_1	−2.47 ± 0.26	−2.74 ± 0.44	0.036	−0.685
S_elb_2	−2.01 ± 0.25	−2.29 ± 0.38	0.018	−0.787
S_elb_3	−2.25 ± 0.12	−2.42 ± 0.28	0.037	−0.678
S_elv_1	−2.56 (0.24)	−2.74 (0.28)	0.008	−0.527
S_elv_4	−2.65 ± 0.27	−2.88 ± 0.39	0.046	−0.652
S_fle_2	−2.51 ± 0.18	−2.71 ± 0.32	0.034	−0.700
S_fle_4	−2.46 (0.55)	−2.74 (0.52)	0.010	−0.835
S_rot_2	−2.51 ± 0.18	−2.71 ± 0.32	0.034	−0.700
S_rot_4	−2.46 (0.55)	−2.74 (0.52)	0.010	−0.835
S_BRA_1	−2.41 ± 0.26	−2.71 ± 0.39	0.016	−0.817
S_BRA_2	−2.24 ± 0.18	−2.51 ± 0.32	0.008	−0.942
S_BRA_3	−2.40 ± 0.24	−2.59 ± 0.29	0.034	−0.698
S_BRA_4	−2.64 ± 0.26	−2.89 ± 0.38	0.030	−0.721
S_TRI_1	−2.37 ± 0.19	−2.59 ± 0.37	0.042	−0.664
S_TRI_2	−2.57 ± 0.17	−2.84 ± 0.21	0.001	−1.330
S_TRI_4	−2.60 ± 0.27	−2.92 ± 0.38	0.008	−0.908
S_AD_2	−2.50 ± 0.18	−2.69 ± 0.25	0.017	−0.800
S_MD_1	−2.43 (0.17)	−2.60 (0.39)	0.019	−0.465
S_MD_4	−2.67 ± 0.25	−2.93 ± 0.39	0.026	−0.737
S_PD_1	−2.50 (0.15)	−2.72 (0.27)	0.003	−0.511
S_PD_2	−2.70 ± 0.19	−2.91 ± 0.24	0.008	−0.916
S_PD_4	−2.71 ± 0.25	−2.97 ± 0.35	0.017	−0.799
S_PM_1	−2.31 (0.36)	−2.54 (0.38)	0.025	−0.526
CCI_AD_PD_2	0.26 (0.03)	0.30 (0.05)	0.008	0.942
CCI_AD_PD_3	0.28 ± 0.03	0.32 ± 0.03	0.001	1.295
CCI_AD_PD_4	0.25 ± 0.04	0.29 ± 0.04	0.003	1.067
CCI_TRL_BB_2	0.27 ± 0.02	0.30 ± 0.04	0.012	0.854
CCI_TRL_BB_3	0.28 ± 0.03	0.32 ± 0.03	0.001	1.173
CCI_TRL_BB_4	0.25 ± 0.05	0.29 ± 0.04	0.008	0.911
CCI_MD_PM_1	0.30 ± 0.02	0.32 ± 0.03	0.008	0.908
CCI_MD_PM_2	0.29 ± 0.01	0.32 ± 0.02	0.003	1.065
CCI_MD_PM_3	0.28 ± 0.02	0.33 ± 0.03	0.004	1.014
CCI_MD_PM_4	0.30 (0.03)	0.34 (0.03)	0.003	1.073

To ensure clarity in tables and figures, feature labels are abbreviated using a consistent format (e.g., S_elb_1). These labels consist of three parts: the feature type, the anatomical descriptor, and the phase number. The feature types are: TD (Trunk Displacement, unit: cm), IC (Interjoint Coordination, unitless correlation coefficient), W (Mechanical Work, unit: J), S (Smoothness, via SPARC, unitless), and CCI (Co-contraction Index, unitless). The anatomical descriptors specify the joint or muscle(s) involved. Joints include: elb (elbow flexion/extension), elv (shoulder elevation/depression), rot (shoulder internal/external rotation), and fle (shoulder flexion/extension). Muscles include: BRA (Brachioradialis), BB (Biceps Brachii), TRL (Triceps Lateral Head), AD (Anterior Deltoid), MD (Middle Deltoid), PD (Posterior Deltoid), and PM (Pectoralis Major). For CCI, pairs (e.g., AD_PD) denote the muscle pair. The numeric subscript (_1 to _4) indicates the task subphase: Phase 1 (Reach), Phase 2 (Transfer to mouth), Phase 3 (Transfer to object), or Phase 4 (Return). Values are mean ± SD (normal) or median (IQR) (non-normal); *p* value represents the P value adjusted using the False Discovery Rate (FDR) method. Cohen’s d represents the effect size for the group difference.

### 2.4 Machine learning algorithm and validation strategy

To systematically evaluate the predictive capability of the models and ensure their generalizability, a two-stage validation strategy was employed: internal validation (based on the model derivation cohort, *n* = 47) and validation on an independent test cohort (*n* = 18).

Within the derivation cohort, eight typical supervised regression algorithms were included for internal comparison: Linear Regression, Ridge Regression, Lasso Regression, Elastic Net Regression, Decision Tree, Extra Trees Regressor, Random Forest Regressor, and Gradient Boosting Regressor. To identify the optimal model and prevent data leakage, all training, tuning, and evaluation procedures were conducted within a rigorous 5-fold cross-validation (CV) pipeline. Crucially, within each of the 5 folds, all preprocessing steps—specifically data standardization (using StandardScaler to achieve zero mean and unit variance) and any feature selection (e.g., RFE)—were fitted only on the training partition (4 folds). The fitted pipeline was then applied to transform both the training partition and the held-out validation partition. Hyperparameter tuning for each algorithm (e.g., the regularization parameter α for Lasso) was also performed within this nested CV structure, using the coefficient of determination (*R*^2^) as the optimization metric. The average performance metrics [*R*^2^, Mean Squared Error (MSE), Mean Absolute Error (MAE)] derived from the held-out validation folds are reported as the model’s internal validation performance.

Based on the internal validation results, the best-performing algorithm (Lasso Regression), which also offered the best balance of predictive accuracy and model interpretability, was identified. Subsequently, a final model of this algorithm was refit using the entire derivation cohort (*n* = 47), with its hyperparameters fixed to the optimal values identified during the cross-validation. Finally, this trained model was evaluated on the completely independent test cohort (*n* = 18). The *R*^2^, MSE, and MAE calculated on this dataset are reported as the model’s independent test performance, providing an unbiased assessment of its generalizability to new patient data.

To enhance model interpretability, feature importance was extracted and ranked based on models with intrinsic feature weights (e.g., linear models and tree-based models). Furthermore, SHAP (SHapley Additive exPlanations) was applied to the representative model to generate both model-level (SHAP summary plots) and instance-level (SHAP force plots) explanations, thereby revealing the direction and magnitude of contributions from key features to the predictions.

## 3 Results

### 3.1 Participant demographic characteristics

A total of 65 stroke patients and 20 healthy controls were included in this study. The stroke patients were divided into a model development cohort (*n* = 47) and an independent test cohort (*n* = 18). As summarized in Table 2, there were no significant differences in demographic or key clinical characteristics, including age, sex, assessed side, type of brain injury, time since injury, and baseline FMA-UL scores, between the two cohorts (all *p* > 0.05).

**Table 2:** Demographic and clinical characteristics of participants.

Variable	Stroke (Development, *n* = 47)	Stroke (Validation, *n* = 18)	*p* value	Healthy controls (*n* = 20)
Age, years, mean ± SD	55.6 ± 8.4	57.1 ± 7.8	0.512	53.8 ± 7.9
Sex (Male / Female)	26 / 21	11 / 7	0.752†	10 / 10
Side of assessed (Right / Left)	33 / 14	12/ 6	0.661†	17 / 3
Type of brain injury, ischemic / hemorrhagic stroke	31 / 16	13 / 5	0.802†	/
Time since brain injury, months, median (IQR)	29.4 (22.5)	27.8 (20.1)	0.887‡	/
FMA-UL, median (IQR)	26.0 (2.5)	25.0 (3.0)	0.456‡	/

† *p*value from Fisher’s exact test; ‡ *p*value from Mann–Whitney U test. *p*values are for comparisons between the Stroke Development and Validation cohorts. FMA-UL, Fugl-Meyer Assessment Upper Limb.

### 3.2 Phase-specific neuromuscular signatures of upper limb dysfunction

To identify phase-specific kinetic impairments that constitute the basis for subsequent machine learning prediction, we first conducted a comprehensive group comparison. The analysis revealed a constellation of phase-specific neuromuscular alterations (Table 1), which delineate the pathophysiology of upper limb dysfunction and provide the foundational feature pool for FMA-UL score prediction.

During the Reach to object phase (Phase 1), dominated by shoulder elevation and elbow extension, patients exhibited impaired motor output stability. This was characterized by significantly decreased smoothness (all *p* ≤ 0.036) of joint moments (S_elb_1, S_elv_1) and multiple muscles (S_PD_1, S_MD_1, S_PM_1). These differences showed moderate to large effect sizes (e.g., S_BRA_1, *d* = −0.817; S_PD_1, *d* = −0.511). Elevated co-contraction of shoulder synergistic pairs (CCI_MD_PM_1; *p* = 0.008, *d* = 0.908) further indicated abnormal stiffness.

In the Transfer to mouth phase (Phase 2), which involves elbow flexion and shoulder elevation, patients demonstrated diminished propulsive power and disrupted coordination. This was evidenced by a 34% reduction in elbow flexion concentric work (W_elb_2, *p* = 0.017, *d* = −0.931) and a 38% decrease in biceps mechanical work (W_BB_2, *p* = 0.017, *d* = −0.805). Coordination between elbow and shoulder motion was impaired in patients (IC_elb_elv_2, *p* = 0.015, *d* = −0.502). This deficit was accompanied by markedly reduced smoothness across the elbow joint (S_elb_2) and multiple muscles (S_fle_2, S_rot_2, S_BRA_2, S_TRI_2, S_AD_2, S_PD_2; all *p* ≤ 0.034), with several showing large effects (e.g., S_TRI_2, *d* = −1.330; S_BRA_2, *d* = −0.942). Patients also showed increased compensatory trunk displacement (TD_2, *p* = 0.036, *d* = 0.680) and elevated co-contraction across all measured muscle pairs (all CCI pairs, *p* ≤ 0.012), all with large effect sizes (*d* ≥ 0.854).

The Transfer to object phase (Phase 3), which requires controlled elbow extension, revealed deficits in eccentric control. Patients showed significantly altered elbow eccentric work (W_elb_3, *p* = 0.017, *d* = 0.866) and altered biceps and triceps work (W_BB_3, *p* = 0.023; W_TRI_3, *p* = 0.036). Elbow-shoulder coordination was significantly degraded (IC_elb_elv_3, *p* = 0.008, *d* = −0.625). Smoothness of the elbow moment (S_elb_3) and brachioradialis force (S_BRA_3) was also compromised (both *p* ≤ 0.037). A pronounced increase in antagonist co-contraction across all muscle pairs (all *p* ≤ 0.004) and increased trunk displacement (TD_3, *p* = 0.012) were observed, with large effect sizes (*d* ≥ 0.682).

Finally, in the Return to start phase (Phase 4), dominated by shoulder abduction, the results highlighted profound compensatory mechanisms. The most salient finding was a large increase in trunk displacement (TD_4, *p* = 0.015, *d* = 0.612). This was associated with altered elbow-shoulder coordination (IC_elb_elv_4, *p* = 0.033) and decreased smoothness across the shoulder joint (S_elv_4) and multiple muscles (S_fle_4, S_rot_4, S_BRA_4, S_TRI_4, S_MD_4, S_PD_4; all *p* ≤ 0.046). Concurrently, all muscle pairs showed elevated co-contraction (all *p* ≤ 0.008), indicating inefficient control and stiffness, with all differences showing large effect sizes (*d* ≥ 0.911).

### 3.3 Model predictive performance and validation

To establish the optimal FMA-UL score prediction model, we systematically compared eight supervised learning regression algorithms. The performance of all models was evaluated on the development cohort using 5-fold cross-validation (internal validation) and on the independent hold-out test cohort (*n* = 18), with detailed results presented in Table 3. The evaluation results indicated that regularized linear models (Lasso, Ridge, ElasticNet) generally outperformed tree-based models (e.g., Random Forest, ExtraTrees) and standard Linear Regression for this task. Among all tested algorithms, the Lasso Regression model demonstrated the best overall performance in both internal validation (*R*^2^ = 0.932) and independent test.

**Table 3:** Comparison of regression model performance on internal validation and independent test cohort.

Model	Internal validation			Independent test		
	*R* ^2^	MSE	MAE	*R* ^2^	MSE	MAE
LinearRegression	0.629	6.672	1.871	0.521	8.945	2.103
Ridge	0.876	2.239	1.184	0.832	2.781	1.315
**Lasso**	**0.932**	**1.201**	**0.799**	**0.881**	**1.802**	**0.954**
ElasticNet	0.921	1.403	0.873	0.865	1.932	1.012
DecisionTree	0.711	5.198	1.452	0.643	6.125	1.698
ExtraTrees	0.893	1.937	0.949	0.818	2.543	1.227
RandomForest	0.832	3.028	1.289	0.794	3.412	1.405
GradientBoosting	0.861	2.527	1.196	0.805	2.874	1.338

Internal validation performance is reported as the mean value from 5-fold cross-validation on the model development cohort (*n* = 47). Independent test performance is evaluated on the independent cohort (*n* = 18). The best performing model (Lasso) is highlighted in bold.

**Figure 2:**
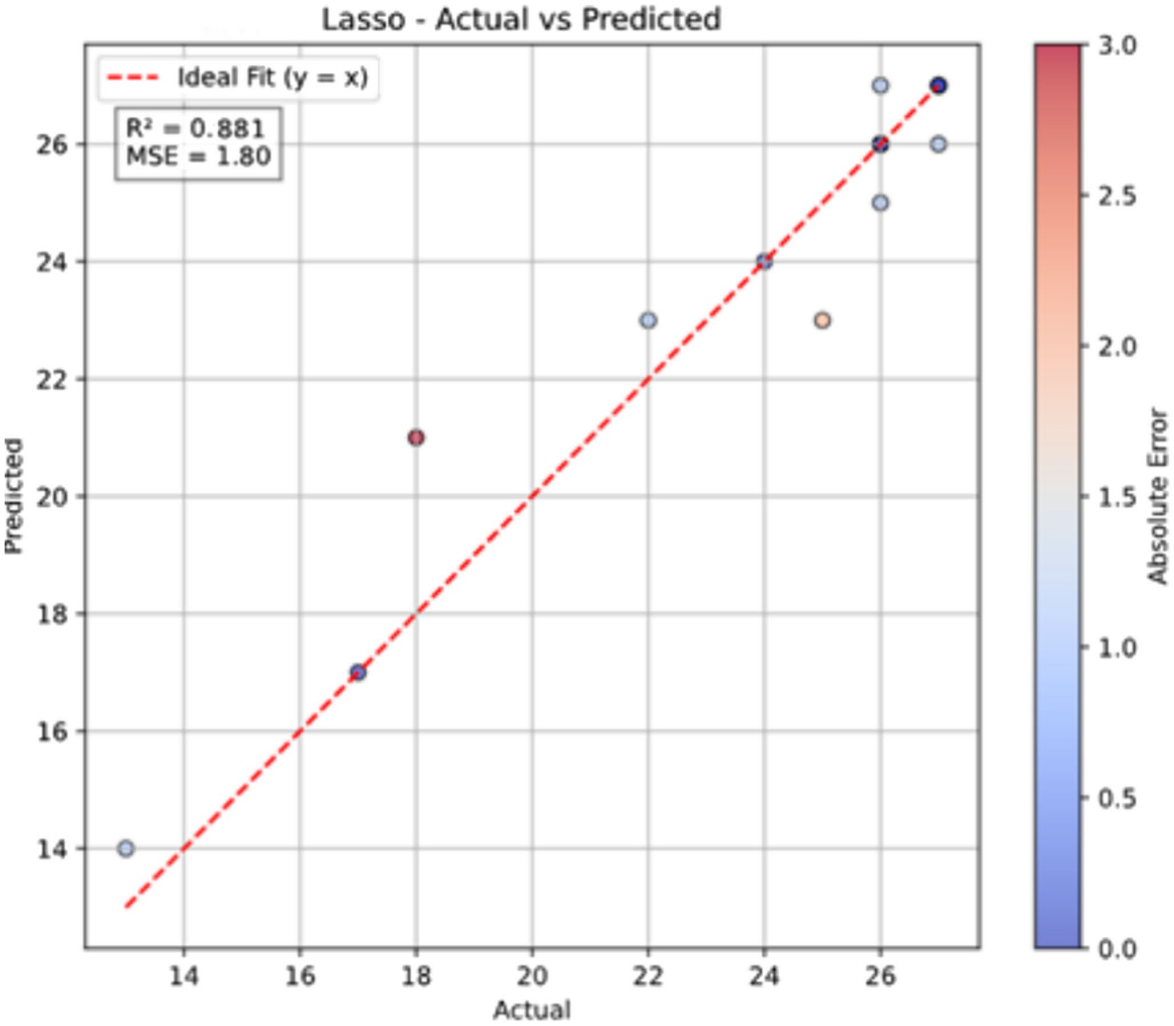
Lasso model performance on the independent test set. The plot shows the correlation between the actual FMA-UL scores and the scores predicted by the model. The dashed red line represents the ideal fit (y = x). The color bar indicates the absolute error for each prediction.

On the more challenging independent test set, the Lasso model achieved the highest coefficient of determination (*R*^2^ = 0.881) and the lowest Mean Squared Error (MSE = 1.802) and Mean Absolute Error (MAE = 0.954). This indicates the model possesses strong generalization capability. Figure 2 illustrates the relationship between the predicted values from the Lasso model and the actual FMA-UL scores on the independent test set. Data points are tightly clustered around the ideal fit line (y = x), visually confirming the model’s high predictive accuracy. Furthermore, the color of the scatter points represents the absolute error, with most points skewed toward blue (low error), further demonstrating that the model’s predictions are reliable at the individual level.

### 3.4 Global model explanation and key feature identification

**Figure 3:**
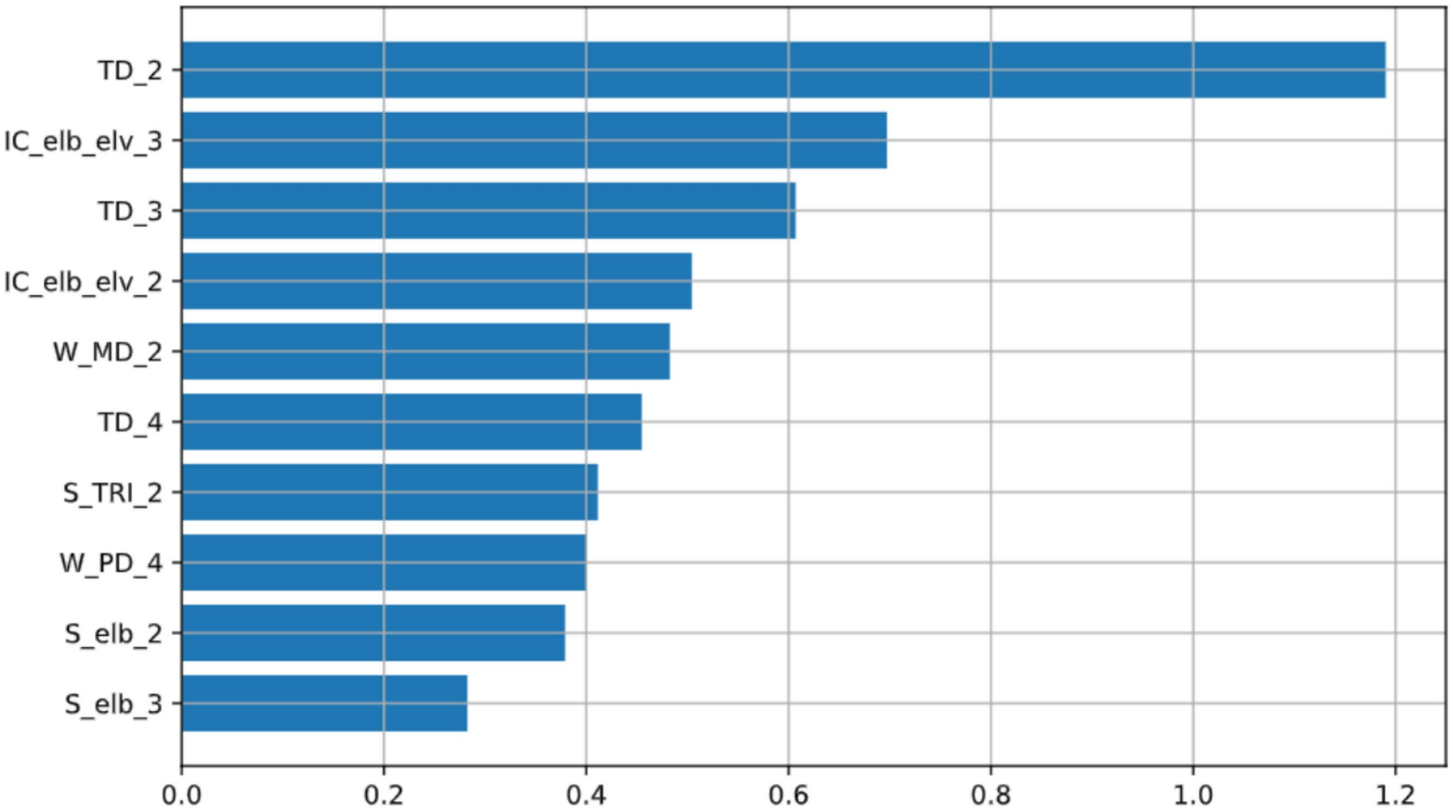
Features with the 10 largest absolute Lasso regression coefficients in the final model. These coefficients characterize the model’s sparse structure and highlight the primary features selected by the algorithm. Feature abbreviations are defined in the note of Table 1.

To understand how the Lasso model makes predictions, we first analyzed its internal structure. Figure 3 displays the 10 features with the largest absolute Lasso regression coefficients (weights) in the final model. As the plot indicates, TD_2 (trunk displacement in phase 2) has the largest coefficient, underscoring its prominent role in the model’s structural composition, followed by IC_elb_elv_3 (elbow–shoulder coordination in phase 3) and TD_3 (trunk displacement in phase 3).

However, while these coefficients define the model’s structure, their rank order can be sensitive to collinearity among correlated features, which is common in biomechanical data. Therefore, to move from reporting the model’s structure (Figure 3) to a more robust analysis of each feature’s practical impact on the prediction, we employed SHAP (SHapley Additive Explanations).

**Figure 4:**
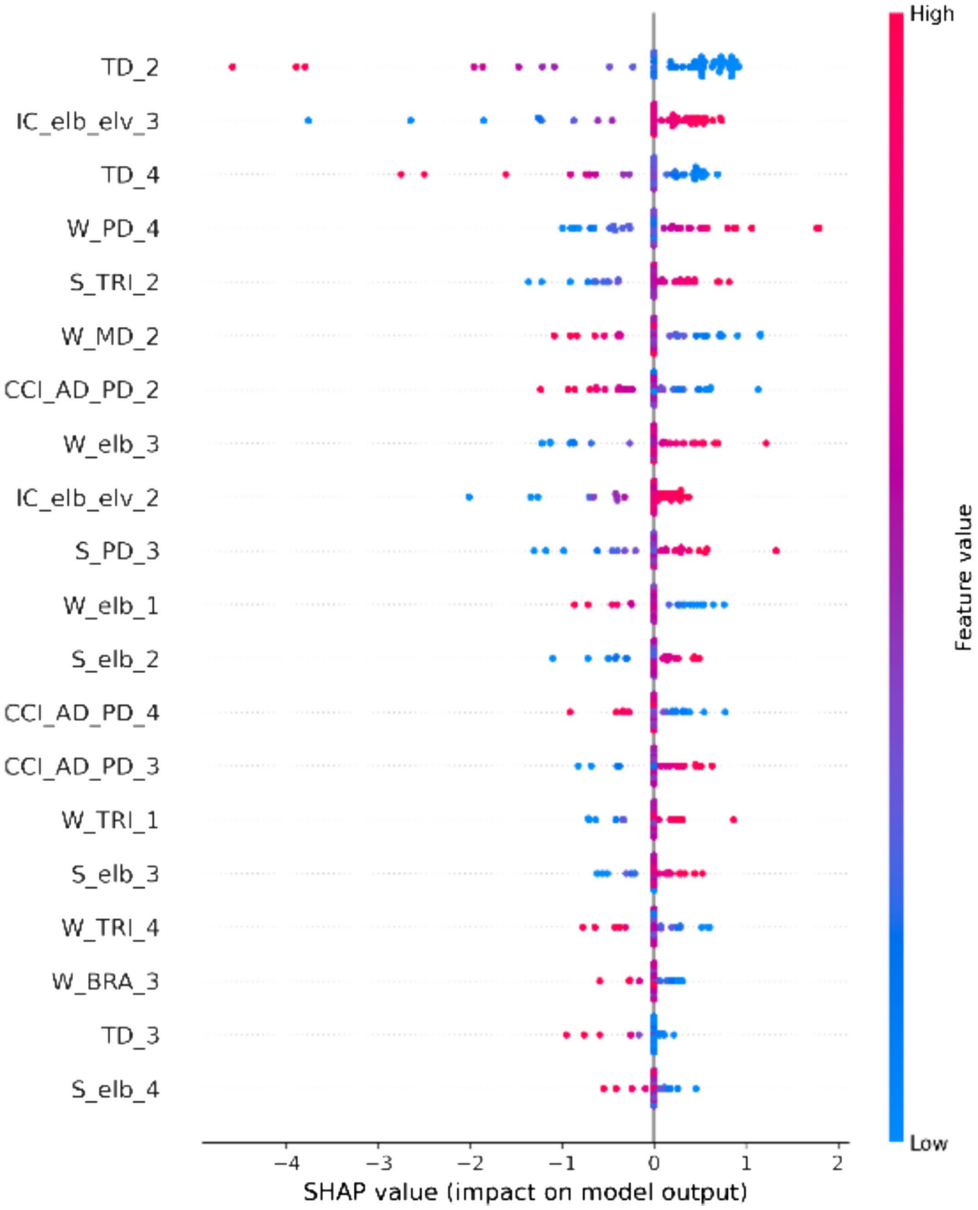
SHAP summary plot ranking features by their mean absolute SHA*p* value. Each dot represents a sample, with its color indicating the feature value (High = Red, Low = Blue) and its x-position indicating the impact on the model prediction. Feature abbreviations are defined in the note of Table 1.

Figure 4 is a SHAP summary plot, which ranks features based on their mean absolute SHAP value and illustrates the impact of each feature’s value (High = Red, Low = Blue) on the model prediction (positive SHAP values push the prediction higher; negative SHAP values push it lower). From the SHAP summary plot, it can be observed that: TD_2 was re-confirmed as the most important feature. Its low values (blue dots) are primarily distributed in the positive SHAP value region, indicating that smaller trunk displacement in phase 2 tends to increase the model’s prediction for the FMA-UL score. IC_elb_elv_3 (phase 3 coordination) and TD_4 (phase 4 trunk displacement) also showed significant impacts. For instance, high values (red dots) for IC_elb_elv_3 are mostly on the positive side, while low values (blue dots) are more skewed to the negative side. TD_4 also exhibited a trend where low values skewed positive. Additionally, high values (red dots) for W_PD_4 (phase 4 posterior deltoid work) and S_TRI_2 (phase 2 triceps smoothness) were also mainly distributed in the positive SHAP value region, suggesting that increases in these kinetic and EMG-related metrics positively contribute to the model’s prediction of functional recovery.

### 3.5 Individualized prediction explanation based on SHAP

**Figure 5:**
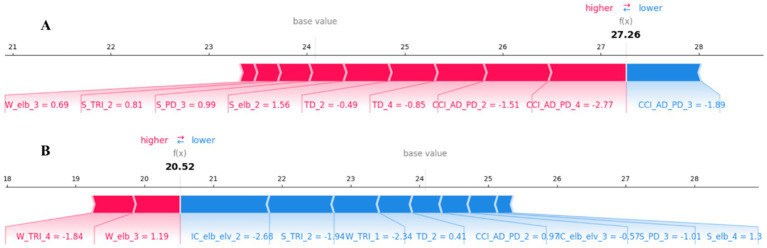
SHAP force plots for local, patient-specific explanations. **(A)** An example prediction [ f(x) = 27.26] that is higher than the base value, showing features that increase the predicted score (red). **(B)** An example prediction [ f(x) = 20.52] that is lower than the base value, showing features that decrease the predicted score (blue). The base value is the average model output over the dataset. Feature abbreviations are defined in the note of Table 1.

In addition to global importance, we also used SHAP force plots to explain how the model makes predictions for specific individual patients. Figure 5 presents two representative individual samples.

In these plots, the f(x) value is the model’s final predicted FMA-UL score for that patient. The base value (approx. 24.2) represents the model’s average predicted score across the entire training dataset. Red arrows represent features that “push” the prediction higher than the base value (i.e., factors increasing the FMA-UL score). Blue arrows represent features that “pull” the prediction lower than the base value (i.e., factors decreasing the FMA-UL score). The length of each arrow represents the magnitude of that feature’s impact (i.e., the magnitude of its SHAP value).

Figure 5A shows a sample with a high predicted score [ f(x) = 27.26]. For this patient, multiple features (red) acted in concert to push the predicted score above the base value, with the most impactful factors including CCI_AD_PD_4, CCI_AD_PD_2, and S_elb_2. The only significant negative factor (blue) was CCI_AD_PD_3. Figure 5B, in contrast, shows a sample with a low predicted score [ f(x) = 20.52]. This prediction is significantly lower than the base value. The main driving factors (blue) for this lower prediction include features such as IC_elb_elv_2, W_TRI_1, and S_TRI_2. Meanwhile, features like W_elb_3 and W_TRI_4 (red) prevented the score from being even lower to some extent. These individualized explanations are crucial for clinically understanding the specific functional impairment patterns of a given patient.

## 4 Discussion

This study successfully developed and rigorously validated an assessment framework that integrates phase-specific, multimodal biomarkers (kinematics and kinetics) derived from wearable sensors (IMU/sEMG) and musculoskeletal modeling to enable a precise, explainable assessment of upper limb function in chronic stroke patients.

A core contribution of this study is the identification of phase-specific neuromuscular signatures of dysfunction. By comparing patients with healthy controls (Table 1), we not only replicated known kinematic deficits, such as impaired inter-joint coordination (IC_elb_elv) and increased compensatory trunk displacement (Repnik et al., 2018), but more importantly, we uncovered the underlying kinetic deficits. For instance, during the ‘Transfer to mouth’ phase (Phase 2), the significant reduction in elbow flexion work (W_elb_2) and biceps work (W_BB_2) (both with large effect sizes, *d* > 0.8) directly quantifies the patient’s failure to generate sufficient propulsive power during a critical ADL. In the ‘Transfer to object’ phase (Phase 3), the altered eccentric elbow work (W_elb_3) revealed deficits in controlled elbow extension. Furthermore, the reduced smoothness of joint moments and muscle forces, alongside elevated co-contraction indices observed across nearly all phases, provides kinetic evidence of inefficient motor control and pathological stiffness post-stroke (Ingram et al., 2021). The key contribution of this study, compared to prior work(Lee et al., 2020; Gomez-Arrunategui et al., 2022), lies in introducing the phase-specific kinetic modeling approach. These phase-specific signatures align closely with clinical observations, uncovering neuromuscular control deficits underlying upper limb dysfunction.

This study demonstrates the robust capability of machine learning to integrate these complex biomarkers to predict clinical function (FMA-UL). Critically, we confirmed the model’s generalizability using an independent test cohort. The Lasso model performed exceptionally in internal cross-validation (*R*^2^ = 0.932) and maintained high accuracy on the completely independent dataset (*R*^2^ = 0.881, MAE = 0.954) (Table 3; Figure 2). The success of the Lasso model, a sparse linear algorithm, suggests that the core information of UL dysfunction may be contained within a sparse but critical set of biomarkers, which benefits model interpretation and future clinical simplification.

By applying explainable AI (SHAP), this study moved beyond “black-box” predictions to reveal the key biomarkers driving model decisions. SHAP analysis (Figure 4) consistently identified trunk displacement (notably TD_2 and TD_3) as the most important predictor. Its direction of impact (negative SHAP contribution) confirms a core tenet of clinical rehabilitation: trunk compensation is a key indicator of poor functional recovery. Low trunk displacement (blue dots in Figure 4) was associated with high FMA-UL predictions (positive SHAP values). These phenomena align with clinical observations of “trunk forward lean compensating for weak shoulder abduction” (Cirstea and Levin, 2000; Burridge et al., 2009; Schwarz et al., 2019), and resonate with Tahmid et al.’s proposition that “EMG-driven models can reveal post-stroke muscle dysfunction” (Tahmid et al., 2022). Furthermore, elbow-shoulder coordination in phase 3 (IC_elb_elv_3) emerged as the second most important feature, highlighting impaired multi-joint synergy as another core deficit (6). Notably, kinetic features such as triceps smoothness in phase 2 (S_TRI_2) and posterior deltoid work in phase 4 (W_PD_4) were also identified as positive contributors (higher values predicted higher FMA-UL scores). This indicates that efficient, smooth muscle force control and the ability to generate sufficient muscle work are hallmarks of functional recovery. This combination of kinematic (compensatory, negative) and kinetic (control quality, positive) features provides a multidimensional perspective on functional impairment. Compared with kinematic parameters alone, kinetic features like muscle mechanical work, co-contraction index, and smoothness better elucidate pathophysiological mechanisms at the neuromuscular level. For instance, an elevated co-contraction index indicates abnormal synergy between antagonistic muscles, a mechanism difficult to capture solely through joint angles (Koh et al., 2023).

These findings have significant clinical translation implications. First, given the reliance on complex biomechanical modeling, the framework is currently better positioned as an in-depth assessment tool for outpatient or rehabilitation centers rather than a rapid bedside screening tool. However, its objective and interpretable nature provides potential for future deployment in telerehabilitation monitoring, overcoming the subjective nature of traditional scales (Stewart and Cramer, 2013). Second, the XAI-identified biomarkers (e.g., TD_2, IC_elb_elv_3) serve as personalized rehabilitation targets. A core advantage of the framework is its ability to generate individualized, explainable reports for clinicians. As shown in Figure 5B, a patient with a low predicted score may be primarily driven by poor coordination (IC_elb_elv_2) and low force smoothness (S_TRI_2), suggesting rehabilitation should focus on coordination training and motor control. TD_2, as the strongest predictor, holds great potential for development as a real-time biofeedback signal to alert patients to compensatory patterns during training, thereby optimizing rehabilitation efficacy.

This study has several limitations. First, while the sample size is statistically adequate, it remains moderate. Second, future research should conduct longitudinal tracking in larger, multi-center cohorts, including patients with a broader range of stroke severity (acute, subacute, and chronic stages and a wider spectrum of FMA-UL scores), to validate these biomarkers and to externally confirm the accuracy, robustness, and explainability of the proposed framework across different hospitals, devices, and clinical workflows. Third, the use of a single HTM task may not fully represent the diversity of upper limb use in daily life. To address this, we recommend expanding the framework to include additional standardized ADL tasks (e.g., reaching, drinking, and object manipulation), which would allow for a more comprehensive evaluation of upper-limb function in stroke patients. Fourth, this study relied on a relatively comprehensive sensor configuration to support the SHAP interpretability analysis. We acknowledge, however, that this configuration has limited feasibility for “rapid assessment” in clinical practice. Future work, guided by the SHAP feature importance results from this study, should explore more streamlined sensor and feature combinations to balance interpretability with clinical utility.

## 5 Conclusion

This study proposed and validated an assessment framework integrating wearable sensors, musculoskeletal dynamics modeling, and explainable machine learning (XAI), to achieve precise assessment of chronic post-stroke upper-limb dysfunction. By analyzing phase-specific multimodal biomarkers from the hand-to-mouth task, this framework successfully uncovered the fundamental neuromuscular kinetic mechanisms underlying chronic post-stroke upper limb dysfunction. We found that trunk compensation during the Transfer to mouth phase (TD_2) and elbow–shoulder coordination during the Transfer to object phase (IC_elb_elv_3) are core elements of functional impairment. The resulting Lasso model not only achieved high-precision prediction of FMA-UL scores but also, critically, demonstrated strong generalizability on an independent test cohort (*R*^2^ = 0.881), thereby enabling an objective and precise quantification of upper limb function in chronic stroke patients. The application of XAI provided clinically-interpretable insights, moving the assessment from how a patient functions to why they are impaired. This work provides a new theoretical basis and powerful technical tools for developing data-driven, objective, and individualized precision rehabilitation strategies.

## Data Availability

The original contributions presented in the study are included in the article/supplementary material, further inquiries can be directed to the corresponding author/s.
